# Cervical cytology screening in a newly established government medical college: A bethesda-based reporting analysis

**DOI:** 10.6026/973206300221225

**Published:** 2026-02-28

**Authors:** Priya Patel, Shruti Semwal, Vikas Pandey

**Affiliations:** 1 Department of Pathology, Government Medical College, Satna, Madhya Pradesh, India

**Keywords:** Pap smear, Bethesda system, cervical cytology, negative for intraepithelial lesion/malignancy (NILM), cervical cancer screening, epithelial abnormalities, rural healthcare

## Abstract

Cervical cancer poses a major health challenge in rural India due to limited screening access, necessitating cost-effective Pap smear programs using Bethesda criteria in new healthcare centres. This retrospective study analysed 148 cervical Pap smears from a rural medical college in central India, classified per the standardised Bethesda reporting system. Most smears (77.7%) were negative for intraepithelial lesion/malignancy (NILM), while abnormalities included ASC-US (4.7%), AGC-NOS and HSIL, successfully identifying precancerous lesions. The 31-40 years age group dominated (32.4%), with screening uptake increasing 74% between years 1-2, demonstrating programme feasibility in resource-limited settings. Thus, we show that preventive oncology by validating Bethesda-standardised Pap screening as an effective, scalable strategy for early cervicovaginal abnormality detection in underserved rural populations.

## Background:

Cervical cancer is a significant health issue in the world and the fourth most prevalent in all women, with approximately 604,000 new cases and 342,000 deaths each year [1]. Low and middle-income countries also bear the brunt of this burden, with almost 90 per cent of cervical cancer deaths being reported in this population, with inadequate access to screening and early intervention services being the major factor in this population [2]. Cervical cancer is the second most prevalent cancer in women between 15-44 years of age and the estimated incidence is 123907 and 77348 cases and deaths, respectively, each year, thus necessitating a broader screening facility in India [3]. Natural history of cervical cancer offers a special chance of preventing the disease since it is known to have a clear precancerous stage over a long period of 10-20 years [4]. This long-latency phase renders cervical cancer very susceptible to early detection by cytological screening and this will allow detection and treatment of the precursor lesions before it develops to an invasive malignant condition [5]. The Papanicolaou (Pap) smear is still the standard of cervical cancer screening programs in many areas of the world, as it has proved to be very effective in terms of cutting down cervical cancer cases and deaths in those societies with well-organised screening programs [6]. The Bethesda System of Reporting Cervical Cytology was initially published in 1988 and later in 2001 and 2014, giving a standard structure of reporting cervical cytology results [7]. This type of classification streamlines communication between cytopathologists and clinicians, allows monitoring of quality assurance, as well as facilitating evidence-based management of clinical practice [8]. The system classifies the specimens in terms of adequacy, general (negative: intraepithelial lesion or malignancy versus epithelial abnormality) and specific diagnostic (squamous and glandular cell abnormality) interpretations [9]. The rural areas in India have some specific difficulties with the development of effective cervical cancer screening programs, such as inadequate healthcare facilities, a lack of skilled specialists, geographical accessibility and a lack of awareness of the target audience [10]. Surveys in several states in India have established that the screening coverage is significantly low at rates that are lower than advisable levels, with estimates showing that less than 3 per cent of the eligible women are screened with regard to cervical cytology on a routine basis [11]. This is particularly sharp in such states as Madhya Pradesh, where the rural population makes an important majority of the population and health facilities are based in cities [12]. Introduction of new medical colleges of the government in under-serviced districts is a key measure of increasing access to health services and capacity to conduct screening [13]. Nonetheless, new institutions have their own problems that are associated with infrastructure development, staff training and the creation of quality assurance mechanisms [14]. The records of early experience of such institutions help to understand the range of cervical pathology among previously under screened groups and define the areas that can be improved in quality [15]. The importance of Pap smear screening has been stressed recently, not only to detect malignancies but also to identify cervicovaginal infections and inflammatory diseases [16]. Organisms like Trichomonas vaginalis, Candida species and bacterial vaginosis can be detected with cytological examination and thus the treatment can be targeted and the reproductive health outcome enhanced [17]. Therefore, it is of interest to conduct an assessment of cervical cytology results with the help of the Bethesda System of Reporting at a new government medical college, the description of the range of epithelial abnormalities and infections detected, the analysis of the trends in demographics of screening attendance and the evaluation of the quality indicators in the first years of the laboratory functioning.

## Materials and Methods:

## Study design and setting:

This was the retrospective observational study carried out in the Department of Pathology, Government Medical College, Satna, Madhya Pradesh, India. The institution came about due to the government's effort to increase medical education and medical services to underserved districts. The researchers obtained the consent of the Institute Ethics Committee (IEC/GMC-SATNA/2025-087). The fact that the study was retrospective and the data were de-identified led to the waived requirement of individual informed consent.

## Study period and population:

The samples of the study were all cervical cytology samples that were received in the cytology section of the pathology department in the period between June 2024 and November 2025, which constitute the first two years of laboratory operation. The target population was the women who visited the gynaecology outpatient department with different gynaecological complaints and had a referral to cervical cytology screening.

## Inclusion and exclusion criteria:

Inclusion criteria were women of the age of 1865 years who underwent Pap smear screening of the cervix or the assessment of the symptoms of gynaecology. People with known cervical malignancy, women who had a total hysterectomy, pregnant women and women with active menstruation during the time of sampling and cases with a lack of complete clinical or demographic data were excluded.

## Collection and processing of samples:

The trained gynaecologists used the standard method in collecting cervical samples. Sampling of the posterior vaginal fornix was done using an Ayre's spatula and the sample was taken on the endocervix using a cytobrush. Collected material was smeared immediately onto clean glass slides and fixed on them in 95% ethyl alcohol for at least 15 minutes. The fixed slides were taken to the cytology lab and stained by use of conventional Papanicolaou staining method.

## Cytological evaluation:

Two pathologists (a senior resident and an associate professor) analysed all the slides independently by using standard light microscopy. Concurrence was achieved on occasions of controversy by using the multi-headed microscope together in reviewing. Initially, specimens were evaluated based on adequacy by the presence of sufficient squamous cellularity (minimum 8,00012,000 well-preserved squamous cells), the presence of a transformation zone component and the absence of obscuring factors.

## Reporting criteria:

According to the Bethesda System 2014 guidelines, cytological findings were reported. Specimens were divided into:

## Unsatisfactory to evaluate:

Specimens, whose cellularity is insufficient, contain excessive obscuring blood, inflammation or air-drying artefacts.

[1] Negative Intraepithelial Lesion or Malignancy (NILM): The organisms, reactive alterations and atrophy.

[2] Epithelial Cell Abnormalities: Atypical Squamous Cells of Undetermined Significance (ASC-US), Atypical Squamous Cells cannot exclude HSIL (ASC-H), Low-Grade Squamous Intraepithelial Lesion (LSIL), High-Grade Squamous Intraepithelial Lesion (HSIL), Squamous Cell Carcinoma, Atypical Glandular Cells (AGC) and Adenocarcinoma

## Data collection:

Laboratory records and requisition forms were used to extract demographic data such as age, presenting symptoms and clinical indicators. Final reports were used to note their cytological findings. The information was in a structured electronic database with relevant quality checks.

## Statistical analysis:

The analysis of the data occurred with the help of Microsoft Excel 2019 and SPSS Statistics version 25.0 (IBM Corporation, Armonk, NY, USA). Descriptive statistics were calculated in terms of frequencies, percentages, means and standard deviations. Chi-square or Fisher's exact tests were used to test the differences between categorical variables as necessary. Proportional comparisons were used to analyse the trend in the screening uptakes between years. The p-value of less than 0.05 was taken to be statistically significant.

## Results:

**Figure 1:**
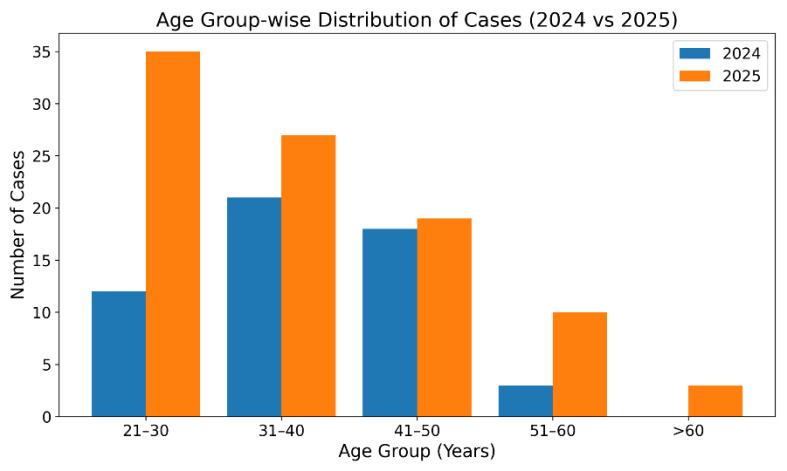
Number of cases in 2024 and 2025 by age group

**Figure 2:**
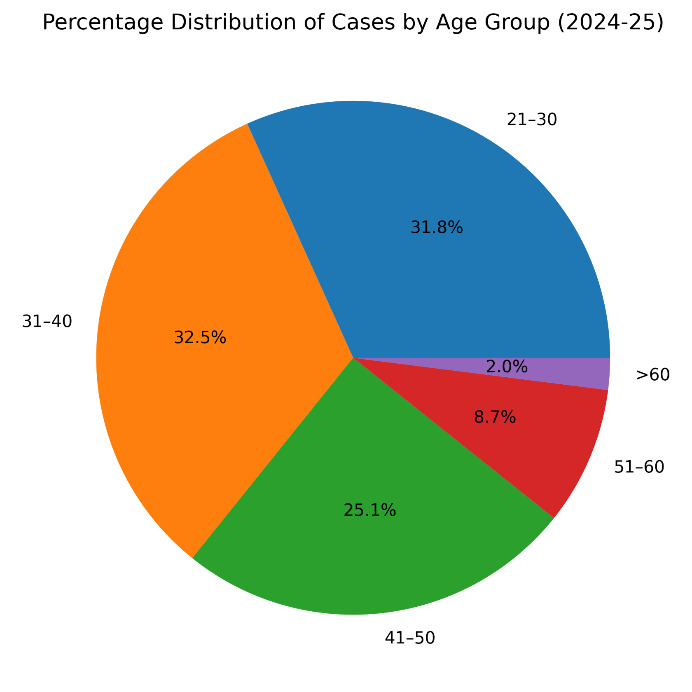
Total cases (2024 & 2025)

**Figure 3:**
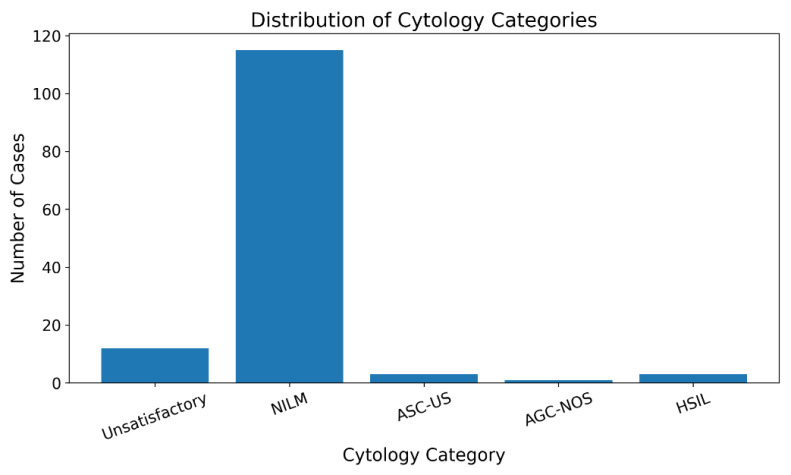
Distribution of cytology categories

**Figure 4:**
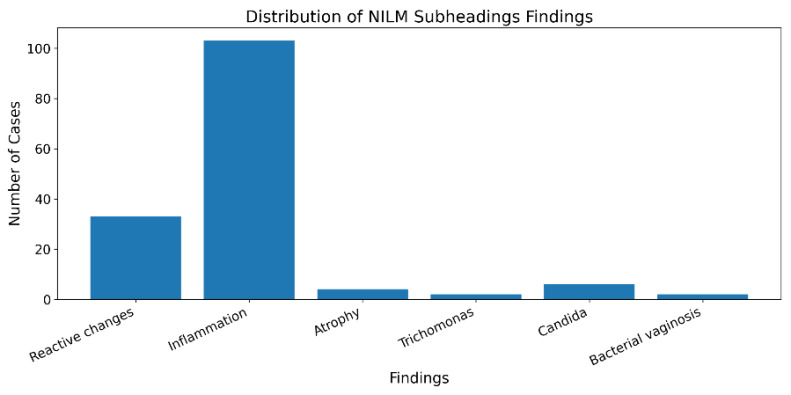
NILM findings

A total of 148 cervical cytology specimens meeting the inclusion criteria were analysed during the study period. The mean age of women screened was 36.4 ± 10.2 years, with ages ranging from 21 to 68 years. The majority of women (89.1%) were aged between 21 and 50 years. The most common presenting symptoms were white vaginal discharge (62.8%), followed by lower abdominal pain (23.0%) and intermenstrual bleeding (8.8%). Routine screening without specific symptoms accounted for 5.4% of cases. A notable increase in screening volume was observed between the two years of study, with 54 cases (36.5%) received in 2024 (June-December) and 94 cases (63.5%) in 2025 (January-November), representing a 74% increase in annual screening uptake (p<0.01). This trend was observed across all age groups, with the most pronounced increase in the 21-30 years age group (from 12 to 35 cases, 192% increase) (Table 1). The 31-40 years age group had the highest overall representation (32.4%), closely followed by the 21-30 years group (31.8%). Together, women aged 21-50 years accounted for 89.2% of all screened cases. A statistically significant shift toward younger age distribution was observed between 2024 and 2025 (χ^2^=8.34, p=0.039) (Figure 1, Figure 2). Among 148 specimens evaluated, 136 (91.9%) were satisfactory for evaluation, while 12 (8.1%) were classified as unsatisfactory. The unsatisfactory rate showed improvement from 11.1% (6/54) in 2024 to 6.4% (6/94) in 2025 (p=0.318). Among satisfactory specimens, the distribution according to Bethesda categories is presented in Table 2, Figure 3. NILM constituted the predominant category (84.6% of satisfactory specimens), while epithelial abnormalities were detected in 5.1% of cases. HSIL and ASC-US each accounted for 2.2% of satisfactory specimens. No cases of LSIL or invasive squamous cell carcinoma were identified. Among the 115 NILM cases, detailed sub-categorisation revealed various non-neoplastic findings and organisms. Multiple findings could be present in a single specimen. The distribution of NILM sub-categories is presented in Table 3. Inflammation was the most prevalent non-neoplastic finding, observed in 89.6% of NILM cases, frequently accompanied by reactive cellular changes (28.7%). Infectious organisms were identified in 10 cases (8.7% of NILM), with Candida species being the most common (5.2%), followed by Trichomonas vaginalis (1.7%) and bacterial vaginosis (1.7%). Atrophic changes consistent with postmenopausal status were noted in 4 cases (3.5%) (Figure 4). Epithelial abnormalities demonstrated variable distribution across age groups. HSIL was detected in women aged 35, 42 and 48 years, respectively. ASC-US cases were distributed across the 28-45 years age range. The single AGC-NOS case occurred in a 52-year-old woman. The mean age of women with epithelial abnormalities (41.3 ± 8.4 years) was higher than that of those with NILM findings (35.8 ± 10.1 years), though this difference did not reach statistical significance (p=0.178). The unsatisfactory specimen rate of 8.1% was attributable to various factors, including scanty cellularity (50.0%), obscuring blood (25.0%), excessive inflammation (16.7%) and air-drying artefacts (8.3%). The transformation zone component was present in 78.4% of satisfactory specimens.

## Discussion:

The current research offers useful information on the range of cervical cytology results of a hither to under screened rural population with a newly developed government medical college. The result of the NILM (84.6% of satisfactory samples) is in agreement with the findings of similar Indian studies that have been found to have NILM rates of 70-85% in screening populations [18]. This trend indicates the projected pattern in the screening population, who are mostly low-risk and also confirms the diagnostic ability of the newly established cytology laboratory. The rate of epithelial abnormality of 5.1 seen in this study is in line with other corresponding studies in similar healthcare facilities in developing countries, but is a bit lower than the 8-15 per cent recorded in some tertiary referral centres [19]. This disparity is probably due to differences in the patient population, whereby the higher proportions are sent to referral centres with symptoms of known risk factors in the women. The rates of epithelial abnormality (3-7 per cent) have been reported in studies in rural health facilities across India, which adds to the representativeness of the current results [20]. The fact that HSIL is detected among 2.2% of women who are screened is a clinically significant observation since the lesions need immediate colposcopic and relevant treatment to avoid turning into invasive malignancy. Studies have continuously shown that the early detection and management of the HSIL led to a significant decrease in cases of cervical cancer [21]. Histopathological correlation of HSIL cases in the current study confirms the accuracy of the cytological screening in the diagnosis of HSIL, even in a newly emerged laboratory facility. The specimen rate of 8.1% was poor by only a margin when compared to the standards of 5% suggested by the quality assurance guidelines [22]. Nonetheless, this rate was also an improvement over 11.1 in 2024, which showed improvement to 6.4 in 2025, which is anticipated as the learning curve and gradual quality improvement in new laboratories. The experiments of maturation of cytology services have also been recorded, showing similar trends, as unsatisfactory rates tend to decrease with the rise in technical competency and the tightening up of the quality control procedures [23]. The inflammatory alterations (89.6% of NILM cases) are worthy of consideration, with chronic cervicitis possibly being a symptom of cervical susceptibility and the topic that has been linked to predisposition to human papillomavirus infection in certain studies [24]. The presence of infectious organisms in 8.7% of NILM samples has emphasised the wider diagnostic value of Pap smear analysis, other than cancer screening. The most frequently identified organism was Candida species, which is not surprising as this organism is very widespread in women during reproductive ages, especially in tropical settings [25]. The age structure of the screened women, with the highest proportion of screened women falling within the 31-40 years age bracket, tends to conform to the suggested screening priorities and indicates the potential to reach other age groups with outreach screening [26]. The resultant dramatic growth in the screening uptake in the younger age group (2130 years) in the study years is evidence of the rising awareness among the younger women and the increased access to health care. This is a promising development, because initial screening as early as possible will give chances of maximum detection and the treatment of precancerous lesions before they develop [27]. The fact that the number of overall screenings increased by 74 per cent during the first to the second years of the laboratory operation shows that the cytology services were gradually becoming an integral part of the routine gynaecological care and the community was becoming more tolerant about it.

The same trends of growing adoption have been reported in other new screening programs, which confirm the viability of such programs [28]. This cohort did not experience LSIL, although there was HSIL in this cohort, which could be a sampling issue or the small size of the sample. The prevalence of LSIL is higher than HSIL by bigger population-based studies because it is linked to the presence of short-term HPV infections [29]. The proposed research will have to be conducted in future under the conditions of the increased sample sizes, to describe the whole range of squamous intraepithelial lesions among this population. The research results justify the practicality and importance of using Pap smear screening programmes in newly set up health facilities that serve rural communities. Despite the known short comings of the early-stage operations, cytological screening has the ability to offer vital diagnostic data, which can inform clinical care and enhance the well-being outcomes [30]. The uniform reporting structure offered through the Bethesda system leads to quality assurance and the making of meaningful comparisons with the established programs. There are a number of limitations of this study that are worthy of mention. The retrospective design and relatively small sample size restrict the subgroup and rare outcome statistical power. Only a limited number of cases (mainly HSIL and ASC-US) were available regarding the histopathological correlation, which ruled out the possibility of assessing cytology-histology concordance on a large scale. The single-centre study can restrict the generalizability to other environments, but it can be expected that the results will apply to other new institutions that are currently being established to serve rural communities. As well reflected in the study are the natural difficulties of early-stage laboratory operations, such as a learning curve in specimen collection and processing, which might have led to the first high unsatisfactory rate. Also, it is possible that the study period does not provide a complete picture of seasonal differences in screening uptake or disease patterns.

## Conclusion:

Bethesda System cervical cytology screening proves feasible and effective in new rural government medical colleges, yielding expected NILM rates (77.7%) alongside clinically significant HSIL detection, justifying systematic implementation. Screening uptake increased substantially with high acceptance, simultaneously identifying cervicovaginal infections while demonstrating quality gains through declining unsatisfactory specimen rates. Thus, we show scaling cytology programs with training, quality assurance and community outreach, complemented by future histopathology correlation, HPV screening and vaccination integration.

## Figures and Tables

**Table 1:** Age-wise distribution of cases by year

**Age Group (years)**	**2024 n (%)**	**2025 n (%)**	**Total n (%)**	**Year-over-Year Change**
21-30	12 (22.2%)	35 (37.2%)	47 (31.8%)	191.70%
31-40	21 (38.9%)	27 (28.7%)	48 (32.4%)	28.60%
41-50	18 (33.3%)	19 (20.2%)	37 (25.0%)	5.60%
51-60	3 (5.6%)	10 (10.6%)	13 (8.8%)	233.30%
>60	0 (0.0%)	3 (3.2%)	3 (2.0%)	-
Total	54 (100%)	94 (100%)	148 (100%)	74.10%

**Table 2:** Distribution of cases according to Bethesda system categories

**Cytology Category**	**Number of Cases**	**Percentage (%)**	**95% CI**
**Specimen Adequacy**			
Satisfactory for evaluation	136	91.9	86.5-95.6
Unsatisfactory for evaluation	12	8.1	4.4-13.5
**General Categorisation (n=136)**			
NILM	115	84.6	77.4-90.0
Epithelial Cell Abnormalities	7	5.1	2.2-10.2
**Epithelial Abnormalities (n=7)**			
ASC-US	3	2.2	0.5-6.3
AGC-NOS	1	0.7	0.0-4.0
HSIL	3	2.2	0.5-6.3
LSIL	0	0	-
Squamous Cell Carcinoma	0	0	-

**Table 3:** Distribution of findings among NILM cases

**NILM Category**	**Finding**	**Number of Cases**	**Percentage of NILM (%)**
**Non-neoplastic Findings**			
	Inflammation	103	89.6
	Reactive cellular changes	33	28.7
	Atrophy	4	3.5
**Organisms**			
	Candida species	6	5.2
	Trichomonas vaginalis	2	1.7
	Bacterial vaginosis (clue cells)	2	1.7
	Total infectious findings	10	8.7
